# Helminth parasites of characiform fishes from farming systems in the states of Acre and Maranhão, Western Amazonia and Northeastern regions, Brazil

**DOI:** 10.1590/S1984-29612026039

**Published:** 2026-08-25

**Authors:** Rayane Duarte, Yuri Costa de Meneses, Marcia Cristina Nascimento Justo, Giovanna Oliveira de Nigris, Gabriela Cristina de França Mendonça, Maralina Torres da Silva, Simone Chinicz Cohen

**Affiliations:** 1 Fundação Oswaldo Cruz, Fiocruz, Instituto Oswaldo Cruz, Laboratório de Helmintos Parasitos de Peixes, Rio de Janeiro, RJ, Brasil; 2 Fundação Oswaldo Cruz, Fiocruz, Instituto Oswaldo Cruz, Programa de Pós-graduação em Biodiversidade e Saúde, Rio de Janeiro, RJ, Brasil; 3 Instituto Federal do Acre, IFAC, Centro de Estudos e Pesquisas em Ambiente, Biodiversidade e Mudanças Climáticas - CEPAB-Clima, Rio Branco, AC, Brasil

**Keywords:** Acanthocephala, Trematoda, fish farm, Monopisthocotyla, fish parasites, Acanthocephala, Trematoda, piscicultura, Monopisthocotyla, parasitos de peixes

## Abstract

Aquaculture has been recognized as one of the main solutions to the growing global food demand, as well as a significant resource for addressing the limitations imposed on wild fisheries. The most important fish species for Brazilian fish farming is *Colossoma macropomum* due to its zootechnical characteristics, economic and cultural importances. In cultivated systems, the presence of parasites can represent a major problem. Under these conditions, parasites of the phyla Acanthocephala and Platyhelminthes are commonly found parasitizing farmed fishes. This study aimed to investigate the helminth fauna and their parasitological indices in cultivated fish farms located in the municipalities of Imperatriz, state of Maranhão and in Rio Branco, state of Acre, Brazil. A total of 1,617 specimens of Monopisthocotyla were collected, and, among the endoparasites, 325 specimens belong to the phylum Acanthocephala and 10 specimens belonging to the class Trematoda were identified. The present study expands the known biodiversity and distribution of helminth parasites in South American aquaculture, providing new records for host-parasite associations and geographic locations in the states of Acre and Maranhão, Brazil.

## Resumo

A aquicultura tem sido reconhecida como uma das principais soluções para a crescente demanda global por alimentos, bem como um recurso significativo para lidar com as limitações impostas à pesca selvagem. A espécie de peixe mais importante para a piscicultura brasileira é *Colossoma macropomum*, devido às suas características zootécnicas e à sua importância econômica e cultural. Em sistemas de cultivo, a presença de parasitos pode representar um grande problema. Nessas condições, parasitos dos filos Acanthocephala e Platyhelminthes são comumente encontrados parasitando peixes de criação. Este estudo teve como objetivo investigar a fauna de helmintos e seus índices parasitológicos em pisciculturas localizadas nos municípios de Imperatriz, no estado do Maranhão, e em Rio Branco, no estado do Acre, Brasil. Foram coletados 1.617 espécimes de Monopisthocotyla e, entre os endoparasitos, foram identificados 325 espécimes pertencentes ao filo Acanthocephala e 10 espécimes pertencentes à classe Trematoda. O presente estudo amplia a biodiversidade e a distribuição conhecidas de helmintos parasitos na aquicultura sul-americana, fornecendo novos registros de associações hospedeiro-parasito e localizações geográficas nos estados do Acre e do Maranhão, Brasil.

## Introduction

Aquaculture has been recognized as one of the main solutions to the growing global food demand, as well as a significant resource for addressing the limitations imposed on wild fisheries. According to the 2023 Aquaculture Report in Union Waters, published by the Brazilian Ministry of Fisheries and Aquaculture (Brasil, 2024), these cultivation systems have driven job creation and become increasingly vital for the local population. In recent years, there has been significant growth in fish production, related to technological advances, infrastructure investments, and growing demand in both the domestic and international markets (Pedroza-Filho et al., 2020a).

The Brazilian Amazon Region stands out for its abundant water and land resources, without significant climatic limitations, and having soils with a high degree of compaction, being considered an ideal location for the development of fish farming activities (Carvalho et al., 2020; Aizawa et al., 2014). Similarly, in the state of Maranhão, fish farming has stood out as a growing activity, favored by the predominantly warm climate, the availability of water, and the vast territorial extension, which contributes to the generation of income and employment in rural areas (Ferreira et al., 2024).

In 2024, the largest growth of the decade occurred in relation to farmed fish production, increasing by 9.21% to 968,745 tons. According to a survey by the Associação Brasileira da Piscicultura – Peixe BR, farmed fish production in the Northern region of the country has remained relatively stable since 2023, reaching 143,190 tons, but the municipality of Rio Branco, in Acre, remained one of the leaders in production (Peixe BR, 2025). In addition, Maranhão ranks sixth nationally in farmed fish production, with 54,500 tons (2024), an increase of 10.9% compared to 2023. Fish farming in Maranhão stands out in the production of both *Oreochromis niloticus* (Linnaeus, 1758) “Tilapia” and native fishes, such as *Colossoma macropomum* (Cuvier, 1816) “Tambaqui” (Peixe BR, 2025).

The most important fish species for Brazilian fish farming is *C. macropomum* “Tambaqui” due to its zootechnical characteristics and its economic and cultural importance, with the potential to consolidate its position in both domestic and international markets, whose meat continues to be increasingly appreciated (Oliveira et al., 2023). *Piaractus brachypomus* (Cuvier, 1818), popularly known as “Pirapitinga”, is another notable species for aquaculture in several tropical countries (*e.g*., Brazil). Its adaptability to different farming systems and its good acceptance in the consumer market make it a species with great zootechnical potential (Kohler, 2022; Santos et al., 2013). In 2023, the “Tambaqui” had a production of 114,000 tons, representing 17.3% of national fish farming (Embrapa, 2025) whereas “Pirapitinga” had a production of 1,748 tons in 2018 (Pedroza Filho et al., 2020b).

In contrast, the Nile tilapia *O. niloticus* stands out as the most cultivated fish in Brazil, representing about 65% of national farmed fish production, equivalent to 579,080 tons in 2023 and 57% of national production in 2019 (Jerônimo et al., 2011; Fernandes‑Neto & Docema, 2025; Peixe BR, 2025). Its prominence is associated with easy reproduction, rusticity, rapid growth, good feed conversion, white and well‑accepted meat, and a well‑defined technological package, making it a commodity of great zootechnical and economic potential for family and commercial farming throughout Brazil.

As a strategy to increase productivity, improve disease resistance, and adapt fish to different environmental conditions, in recent years Brazil has been investing heavily, especially in the fish farming sector, in the creation of hybrid fish, resulting from controlled crossbreeding (Hashimoto et al., 2012). In this context, the hybrids “Tambatinga” (*Colossoma macropomum* ♀ x *Piaractus brachypomus* ♂) and “Tambacu” (*Piaractus mesopotamicus* ♂ x *Colossoma macropomum* ♀) stand out as the most cultivated at a national level (Silva et al., 2018). In 2018, “Tambatinga” and “Tambacu” had a production of 40,000 tons in Brazil (Pedroza-Filho et al., 2020b).

In cultivated systems, the presence of parasites can represent a major problem. Under these conditions, parasites of the phyla Acanthocephala and Platyhelminthes, such as members of the class Monopisthocotyla, as well as members of the class Trematoda are commonly found parasitizing farmed fish (Buchmann, 2022).

This study aimed to investigate the helminth fauna and their parasitological indices in *C. macropomum*, *P. brachypomus*, “Tambatinga” (*Colossoma macropomum* ♀ x *Piaractus brachypomus* ♂) and “Tambacu” (*Piaractus mesopotamicus* ♂ x *Colossoma macropomum* ♀) cultivated in fish farms located in the Brazilian states of Acre and Maranhão. Additionally, the survey presents a literature review on helminth parasites of fishes from cultivated systems in South America.

From a broader perspective, Table 1 compiles records of helminth species (Monopisthocotyla, Trematoda, and Acanthocephala) across various fish species in South American cultivated systems, categorized by parasite species, host, country of occurrence, and the original bibliographic references.

**Table 1:** Helminth species (Monopisthocotyla, Trematoda, and Acanthocephala) parasites of fishes from cultivated systems in South America.

**Species of parasites**	**Hosts**	**Country**	**References**
**Monopisthocotyla (Dactylogyridae)**			
*Amphocleithrum paraguayensis* Price & Romero, 1969	*Pseudoplatystoma reticulatum x P. corruscans*	Brazil	Jerônimo et al. (2016)
*Apedunculata discoidea* Cuglianna, Cordeiro & Luque, 2009	*Prochilodus lineatus* (Valenciennes, 1837)	Brazil	Cuglianna et al. (2009)
*Anacanthorus penilabiatus* Boeger, Husak & Martins, 1995	*Colossoma macropomum* (Cuvier, 1816); *Piaractus brachypomus* (Cuvier, 1818); *Piaractus mesopotamicus* (Holmberg, 1887); *Colossoma macropomum x Piaractus mesopotamicus; Piaractus mesopotamicus x Piaractus brachypomus*	Brazil	Pamplona-Basilio et al. (2001); Cohen & Kohn (2009); Jerônimo et al. (2020)
Anacanthorus spatulatus* Kritsky, Thatcher & Kayton, 1979	*Colossoma macropomum* (Cuvier, 1816);	Brazil; Peru	Lizama et al. (2007b); Morais et al. (2009); Santos et al. (2013); Godoi et al. (2012);Soberon et al. (2014); Dias et al. (2015a); Morey et al. (2026)
	*Colossoma macropomum x Piaractus brachypomus*		
	*Piaractus mesopotamicus* (Holmberg, 1887)		
*Cichlidogyrus halli* (Price & Kirk, 1967) Paperna, 1979	*Oreochromis niloticus* (Linnaeus, 1758)	Brazil	Jerônimo et al. (2011)
*Cichlidogyrus sclerosus* Paperna & Thurston, 1969	*Oreochromis niloticus* (Linnaeus, 1758)	Brazil	Lizama et al. (2007a); Jerônimo et al. (2011)
*Cichlidogyrus thurstonae* Ergens, 1981	*Oreochromis niloticus* (Linnaeus, 1758)	Brazil	Jerônimo et al. (2011)
*Cichlidogyrus tilapiae* Paperna, 1960	*Oreochromis niloticus* (Linnaeus, 1758)	Brazil	Pantoja et al. (2012)
*Dactylogyrus extensus* Mueller & Van Cleave, 1932	*Cyprinus carpio* (Linnaeus, 1758)	Brazil	Santos et al. (2017)
*Dactylogyrus minutus* Kulwiec, 1927	*Cyprinus carpio* (Linnaeus, 1758)	Brazil	Santos et al. (2017)
*Demidospermus paravalenciennsi* Gutierrez & Suriano, 1992	*Synodontias clarias* (Linnaeus, 1758)	Argentina	Gutiérrez & Suriano (1992); Kritsky & Gutiérrez (1998)
*Linguadactyloides brinkmanni* Thatcher & Kritsky, 1983	*Colossoma macropomum* (Cuvier, 1816);	Brazil	Aragort et al. (2002); Morais et al. (2009); Dias et al. (2012, 2015a)
	*Colossoma macropomum X Piaractus brachypomus*		
*Mymarothecium boegeri* Cohen & Kohn, 2005	*Colossoma macropomum* (Cuvier, 1816); *Colossoma macropomum x Piaractus brachypomus*	Brazil	Cohen & Kohn (2005, 2009); Morais et al. (2009); Santos et al. (2013); Dias et al. (2015a)
*Mymarothecium viatorum* Boeger, Piasecki & Sobecka, 2002	*Piaractus brachypomus*(Cuvier, 1818); *Piaractus mesopotamicus* (Holmberg, 1887); *Piaractus mesopotamicus* x *Piaractus brachypomus*; *Serrasalmus spilopleura* (Kner, 1858)	Brazil; Peru	Kritsky et al. (1996); Cohen & Kohn (2005, 2009); Jerônimo et al. (2020)
*Notozothecium euzeti* Kritsky, Boeger & Jégu, 1996	*Colossoma macropomum* (Cuvier, 1816); *Serrasalmus altispinis* Merckx, Jégu & Santos, 2000	Brazil	Cohen & Kohn (2009); Dias et al. (2015b); Morey et al. (2017); Morey & Malta (2018)
*Notozothecium janauachensis* Belmont-Jégu, Domingues & Martins, 2004	*Colossoma macropomum* (Cuvier, 1816); *Colossoma macropomum x Piaractus brachypomus*	Brazil	Cohen & Kohn (2009); Morais et al. (2009); Dias et al. (2015a); Jerônimo et al. (2020); Morey et al. (2026)
*Pavanelliella pavanellii* Kritsky & Boeger, 1998	*Pseudoplatystoma corruscans* (Spix & Agassiz, 1829); *Calophysus macropterus* (Lichtenstein, 1819); *Pimelodus maculatus* (Lacepède, 1803)	Brazil	Kritsky & Boeger (1998); Brasil-Sato & Pavanelli (2000); Takemoto et al. (2009)
*Rhinonastes pseudocapsaloideum* Kritsky, Thatcher & Boeger, 1988	*Prochilodus nigricans* Spix & Agassiz, 1829	Peru	Mathews et al. (2013)
*Rhinoxenus piranhus* Kritsky, Boeger & Thatcher, 1988	*Serrasalmus altispinnis* Merckx, Jégu & Santos, 2000; *Serrasalmus altuvei* (Ramírez, 1965)	Brazil	Morey & Malta (2018); Leão et al. (1991)
*Scutogyrus longicornis* (Paperna & Thurston, 1969) Pariselle & Euzet, 1995	*Oreochromis niloticus* (Linnaeus, 1758)	Brazil	Jerônimo et al. (2011)
*Tereancistrum curimba* Lizama, Takemoto & Pavanelli, 2004	*Prochilodus lineatus* (Valenciennes, 1837)	Brazil	Lizama et al. (2006)
*Tereancistrum ornatus* Kritsky, Thatcher & Kayton, 1980	*Prochilodus lineatus* (Valenciennes, 1837)	Colombia	Dias et al. (2017)
*Trinibaculum altiparanae* Abdallah, Azevedo & Silva, 2013	*Prochilodus lineatus* (Valenciennes, 1837); *Astyanax altiparanae* Garutti & Britski, 2000	Brazil	Leite et al. (2018); Abdallah et al. (2013)
*Vancleaveus fungulus* Kritsky, Thatcher & Boeger, 1986	*Pseudoplatystoma tigrinum* (Valenciennes, 1840); *Pseudoplatystoma reticulatum x P. corruscans*	Brazil	Kritsky et al. (1986); Jerônimo et al. (2016)
**Trematoda**			
Dadaytrema oxycephalum (Diesing, 1836)	*Piaractus mesopotamicus* (Holmberg, 1887)	Argentina	Furlan et al. (2025)
**Acanthocephala**			
*Neoechinorhynchus buttnerae* Golvan, 1956	*Colossoma macropomum* (Cuvier, 1818)	Brazil	Malta et al. (2001); Jerônimo et al. (2017); Lourenço et al. (2017); Aguiar et al. (2018); Pereira & Morey (2018); Valladão et al. (2020);Perez Pedroti et al. (2024)

* The original description named this species after its spatula-shaped accessory piece (in Latin, *spatulatus*, without the letter "h"). However, the original article misspelled the name as *A. spathulatus* with an 'h', likely due to a typographical error. Based on the original etymology, the authors from the present study confirm that the correct spelling is *A. spatulatus*.

## Material and methods

Between September 2017 and May 2019, nine specimens of *C. macropomum* were commercially obtained from a fish farm located in the municipality of Imperatriz, state of Maranhão, Brazil (5°25'56" S 47°28'20" W). Additionally, between May and June 2023 and March and September 2024, 54 specimens of four species were commercially obtained from a fish farm located in the municipality of Rio Branco, state of Acre, Brazil (9°58'19" S 67°47'54" W). These specimens included 27 of *Colossoma macropomum* “Tambaqui”, 12 of *Piaractus brachypomus* “Pirapitinga”, 11 specimens of the hybrid *Colossoma macropomum* ♀ x *Piaractus brachypomus* ♂ “Tambatinga”, and four of hybrid *Piaractus mesopotamicus* ♂ x *Colossoma macropomum* ♀ “Tambacu”.

Gills were removed and placed in vials containing hot water (*c*. 65 ºC) and were vigorously shaken to detach the parasites from the gill filaments. Absolute ethanol was added to reach a concentration of 70%. The internal organs of the fish specimens were removed and preserved in ethanol 70%. The vials of the gills and viscera were sent to the “Laboratório de Helmintos Parasitos de Peixes, Instituto Oswaldo Cruz, Fiocruz”, Rio de Janeiro, Brazil.

In the laboratory, the samples are then examined under a stereomicroscope for helminths. Monopisthocotylan specimens were mounted unstained in Hoyer’s medium for the study of the sclerotized parts; trematodes and acanthocephalans were stained with Langeron’s carmine, dehydrated in alcohol series, clarified in clove oil or Beechwood creosote, and mounted in Canada balsam as permanent slides (adapted from Amato et al., 1991). These specimens were studied on a Zeiss Axioskop microscope with differential interference contrast (DIC) apparatus and an Olympus™ BX 41 microscope with phase contrast.

Parasitological indices, such as prevalence (P%), mean intensity (MI), and mean abundance (MA) were calculated according to Bush et al. (1997) and are presented as mean followed by standard deviation (SD). The classification of parasitic species followed the core-satellite distinction proposed by Bush & Holmes (1986), in which species are categorized based on their prevalence in the examined hosts, with those having values greater than 66.6% considered core, those between 33.3% and 66.6% considered secondary, and those below 33.3% considered satellite.

## Results

A total of 1,617 specimens of Monopisthocotyla belonging to the Dactylogyridae were collected from the gills and identified as: *Anacanthorus penilabiatus* (eight specimens), *Anacanthorus reginae* Boeger & Kritsky, 1988 (four specimens), *Anacanthorus spatulatus* (945 specimens), *Annulotrematoides parisellei* Cohen, Kohn & Boeger, 2012 (six specimens), *Linguadactyloides brinkmanni* (18 specimens), *Mymarothecium boegeri* (24 specimens), *Mymarothecium iiapensis* Morey, Aliano & Grandez, 2019 (128 specimens), *Mymarothecium tantaliani* Cayulla-Quispe, Mondragón-Martínez, Rojas De Los Santos, Garcia-Candela, Babilonia-Medina & Martínez-Rojas, 2021 (169 specimens), *Mymarothecium viatorum* (82 specimens), *Notozothecium euzeti* (three specimens), and *Notozothecium janauachensis* (230 specimens).

Among the endoparasite specimens collected in the viscera content, 326 specimens belonged to the phylum Acanthocephala, and which were identified as *N. buttnerae*; 10 specimens belonged to the class Trematoda which were identified as *D. oxycephalum*.

The parasitological indices of the helminth fauna found in the four host species from fish farms in Rio Branco (Acre State, Brazil) are presented in Table 2 and organized by host species. This table details the Monopisthocotyla species identified, including the total number of parasitized (HP), total of helminth specimens (TH), prevalence (P%), mean intensity (MI), and mean abundance (MA), with their respective standard deviations (± SD). Furthermore, these results document significant biological findings, including new host records and new geographic location records for the region.

**Table 2:** Helminth parasites of four host species obtained from fish farms located in Rio Branco, Acre State, Brazil. HP – total of hosts parasitized; TH – total of helminths collected; P% – prevalence; MI – mean intensity; MA – mean abundance; SD – standard deviation

***Piaractus brachypomus (Pirapitinga)***					
**Monopisthocotyla**	**HP**	**TH**	**P%**	**MI ± SD**	**MA ± SD**
*Anacanthorus reginae* ^1^	2	3	16,7%	1,5 ± 0,7	0,3 ± 0,6
*Anacanthorus spatulatus*	10	26	83,3%	2,6 ± 2,8	2,2 ± 2,7
*Mymarothecium boegeri* ^1^	5	7	41,7%	1,4 ± 0,9	0,6 ± 0,9
*Mymarothecium tantaliani* ^1, 2^	3	5	25%	1,6 ± 0,6	0,4 ± 0,8
*Mymarothecium iiapensis* ^1, 2^	1	1	8,3%	1,0	0,1 ± 0,3
*Mymarothecium viatorum*	11	72	91,7%	6,5 ± 4,4	6,0 ± 4,6
**Trematoda**					
*Dadaytrema oxycephalum*	1	9	8,3%	9,0	0,8 ± 2,6
**Acanthocephala**					
*Neoechinorhynchus buttnerae* ^1^	1	1	8,3%	1,0	0,1 ± 0,3
***Colossoma macropomum* x *Piaractus brachypomus* (Tambatinga)**					
**Monopisthocotyla**	**HP**	**TH**	**P%**	**MI ± SD**	**MA ± SD**
*Anacanthorus penilabiatus* ^1^	2	6	16,7%	3,0 ± 2,8	0,5 ± 1,5
*Anacanthorus spatulatus*	10	91	90,9%	9,1 ± 0,6	8,3 ± 10,8
*Mymarothecium boegeri*	4	10	36,4%	2,5 ± 0,6	0,9 ± 1,3
*Mymarothecium viatorum* ^1^	2	10	18,2%	5,0 ± 4,2	0,9 ± 2,4
*Notozothecium janauachensis*	1	1	9,1%	1,0	0,1 ± 0,3
**Trematoda**					
*Dadaytrema oxycephalum* ^1^	1	1	9,1%	1,0	0,1 ± 0,3
**Acanthocephala**					
*Neoechinorhynchus buttnerae*	1	7	9,1%	7,0	0,6 ± 2,1
***Piaractus mesopotamicus* x *Colossoma macropomum* (Tambacu)**					
**Monopisthocotyla**	**HP**	**TH**	**P%**	**MI ± SD**	**MA ± SD**
*Anacanthorus spatulatus* ^1^	4	138	100%	34,5 ± 33,2	34,5 ± 33,2
*Mymarothecium boegeri* ^1^	1	1	25%	1,0	0,3 ± 0,5
***Colossoma macropomum* (Tambaqui)**					
**Monopisthocotyla**	**HP**	**TH**	**P%**	**MI ± SD**	**MA ± SD**
*Anacanthorus penilabiatus*	2	2	7,4%	1,0	0,1 ± 0,3
*Anacanthorus reginae* 1	1	1	3,7%	1,0	0,04 ± 0,2
*Anacanthorus spatulatus*	26	663	96,3%	25,5 ± 35,3	24,6 ± 34,9
*Linguadactyloides brinkmanni*	5	18	18,5%	3,6 ± 2,6	0,7 ± 1,8
*Mymarothecium boegeri*	3	6	11,1%	2,0 ± 1,7	0,2 ± 0,8
*Mymarothecium iiapensis* 2	7	124	25,9%	17,7 ± 24,4	4,6 ± 24,4
*Mymarothecium tantaliani* ^2^	14	164	51,9%	11,7 ± 21,3	6,1 ± 16,2
*Notozothecium euzeti*	2	3	7,4%	1,5 ± 0,7	0,1 ± 0,7
*Notozothecium janauachensis*	12	226	44,4%	18,8 ± 32,4	8,4 ± 32,4

1 New host record;

2 New geographic location record.

For the samples collected in Imperatriz (Maranhão State, Brazil), the parasitological data regarding *C. macropomum* are summarized in Table 3. Following the same structure as the previous data, it highlights the infestation patterns through indices of prevalence (P%), the mean intensity and abundance (MI ± SD; MA ± SD) based on the total number of specimens (NT) for each parasite group.

**Table 3:** Helminth parasites of *Colossoma macropomum* from fish farm located in Imperatriz, Maranhão State, Brazil. HP – total of hosts parasitized; TH – total of helminths collected; P% – prevalence; MI – mean intensity; MA – mean abundance; SD – standard deviation

***Colossoma macropomum (Tambaqui)***					
**Monopisthocotyla**	**HP**	**TH**	**P%**	**MI ± SD**	**MA ± SD**
*Anacanthorus spatulatus*	6	27	66,7%	4,5 ± 3,2	3,0 ± 3,4
*Annulotrematoides parisellei* 1	2	6	22,2%	3,0 ± 1,4	0,7 ± 1,4
*Mymarothecium iiapensis*	1	3	11,1%	3,0	0,3 ± 1,0
*Notozothecium janauachensis*	1	3	11,1%	3,0	0,3 ± 1,0
**Acanthocephala**					
*Neoechinorhynchus buttnerae*	4	318	44,4%	79,5 ± 147,0	35,3 ± 99,3

1 New host record.

## Discussion

This study revealed new records of helminths of the classes Monopisthocotyla and Trematoda and of the phylum Acanthocephala parasitizing freshwater cultivated fishes from the states of Acre and Maranhão, Brazil.

Species of *Mymarothecium* Kritsky, Boeger & Jégu, 1996 are commonly found infesting characiform fishes (Kritsky et al., 1996). *Mymarothecium boegeri* was originally reported parasitizing *C. macropomum* and *C. macropomum* ♀ × *P. brachypomus* ♂ (Table 1). In the present study, this species is reported for the first time parasitizing *P. brachypomus* and *P. mesopotamicus* ♂ x *C. macropomum* ♀. *Mymarothecium tantaliani* and *M. iiapensis* were originally described parasitizing *C. macropomum* collected from Peru (Cayulla-Quispe et al., 2021; Morey et al., 2019) and are now reported here for the first time parasitizing *P. brachypomus*. Similarly, *M. viatorum*, which has been reported parasitizing *P. brachypomus*, *P. mesopotamicus*, *P. mesopotamicus* x *P. brachypomus*, and *S. spilopleura* from different localities in Brazil (Table 1), is now recorded for the first time parasitizing the gills of *C. macropomum* x *P. brachypomus*. In addition, the finding of *M. tantaliani* and *M. iiapensis* in *P. brachypomus* in the cultivated system in Brazil represents a new record of geographic distribution (Table 2).

*Anacanthorus* Mizelle & Price, 1965 comprises species that are exclusively parasites of Neotropical characiform fishes (Silva et al., 2022). *Anacanthorus penilabiatus* was recorded in the gills of *C. macropomum*, *P. brachypomus*, *P. mesopotamicus*, and the hybrids “Tambacu” (*P. mesopotamicus* ♂ x *C. macropomum* ♀) and “Patinga” (*P. mesopotamicus* x *P. brachypomus*) from different localities in cultivated systems in Brazil (Table 1). In this study, this species is reported for the first time from the host “Tambatinga” (*C. macropomum* ♀ x *P. brachypomus* ♂) (Table 2), representing a new host record for the species. *Anacanthorus spatulatus* has been described parasitizing *C. macropomum*, *P. brachypomus*, *P. mesopotamicus*, *Potamorhina latior* (Spix & Agassiz, 1829) and the hybrid *C. macropomum* ♀ x *P. brachypomus* ♂ from different localities in Brazil, Venezuela and Peru (Table 1) (Kritsky et al., 1979; Fischer et al., 2003; Centeno et al., 2004; Oliveira & Tavares-Dias, 2016). In the present study, a new host is recorded, with the occurrence of this species parasitizing the “Tambacu” hybrid (*P. mesopotamicus* ♂ x *C. macropomum* ♀) (Table 1).

Similarly, *A. reginae* originally described by Boeger & Kritsky (1988) from *Pygocentrus nattereri* (= *Serrasalmus nattereri*) (Characiformes, Serrasalmidae) obtained from Brazilian Amazon is reported for the first time parasitizing the gills of *C. macropomum* and *P. brachypomus*.

*Annulotrematoides parisellei* was originally reported parasitizing *Salminus brasiliensis* (Cuvier, 1816) and later in *Salminus hilarii* Valenciennes, 1850 from different localities in Brazil (Cohen et al., 2012; Brandão et al., 2013), and in this study is reported for the first time parasitizing the gills of *C. macropomum* and was only found in fishes from cultivated systems from Maranhão State.

The discovery of new hosts highlights the importance of ongoing studies to understand the distribution and adaptability of this parasite in different fish species. This scenario is corroborated by the study by Neves et al. (2020), which confirms the idea that the diversity and adaptability of monopisthocotylans are greater than previously recognized, highlighting the importance of continuous monitoring to detect changes in their distribution and impact.

This study also presented new records of the geographic distribution of monopisthocotylan species, like *M. iiapensis* and *M. tantaliani*, both originally described in the Peruvian Amazon, are reported here for the first time from fish farming systems in Brazil (Tables 2, 3). However, their occurrence varied between the analyzed regions: in the state of Maranhão, only *M. iiapensis* was identified, exclusively parasitizing *C. macropomum*. In contrast, in the state of Acre, both species were recorded across a broader range of hosts, including *C. macropomum*, *P. mesopotamicus* and the hybrids *C. macropomum* ♀ x *P. brachypomus* ♂ and *P. mesopotamicus* ♂ x *C. macropomum* ♀.

Among the species identified, *A. spatulatus* stood out as the most abundant in the state of Acre and Maranhão, with an infestation intensity of up to 194 individuals in a single host of *C. macropomum* in the state of Acre. These results indicate that the farming environments in Acre and Maranhão offer highly favorable conditions for the reproduction and spread of these ectoparasites. *Anacanthorus spatulatus* consistently showed the highest prevalence and parasitic intensity, classifying it as a core species. Its dominance suggests that *A. spatulatus* has a strong and successful adaptation to the hosts studied. Therefore, high prevalence indicates an efficient transmission mechanism and/or a high exposure rate, while high intensity suggests a superior ability to establish, survive, and persist in the environment. This postulate could explain the presence of this species in large numbers in the environments studied. This predominance of *A. spatulatus* parasitizing the gills of cultivated fish was observed in different studies (Negreiros & Tavares-Dias, 2019; Silva et al., 2022; Morey et al., 2026).

In the Rio Branco fish farm, *M. viatorum* presented the highest prevalence, mean intensity, and mean abundance values in *P. brachypomus* (Table 2). This pattern underscores the host specificity of *M. viatorum* for *Piaractus* spp., reinforcing previous findings from different South American and Amazonian basins (Table 1) (Oliveira & Tavares-Dias, 2016). Cohen & Kohn (2005, 2009) highlighted that this species has high direct transmission efficiency, favored by farming environments with high stocking densities.

*Mymarothecium boegeri* has a distribution pattern that places it in the category of “secondary species” in the *C. macropomum* ♀ x *P. brachypomus* ♂ parasite community (36.36% prevalence) and as a “satellite species” in *P. mesopotamicus* ♂ x *C. macropomum* ♀ (25% prevalence). Although its prevalence in “Tambatinga” (*C. macropomum* ♀ x *P. brachypomus* ♂) places it slightly above the rarity threshold, the most notable feature is the consistency of low intensities and mean abundances in both hybrids.

From a comparative perspective, the data available for *N. janauachensis* parasitizing “Tambaqui” in cultivation in Acre reveal an infection pattern of great ecological and sanitary relevance. With a prevalence of 44.44% and a significant average abundance, this species falls into the category of “secondary species”. *Notozothecium janauachensis* is recognized as typical of “Tambaqui”, both in natural environments and in fish farming, according to Belmont-Jégu et al. (2004) and corroborated by subsequent studies (Table 1) (Belmont‑Jégu et al., 2004; Cohen & Kohn, 2009; Morais et al., 2009; Oliveira & Tavares-Dias, 2016). In contrast, *N. euzeti* and *A. reginae* had the lowest prevalence and intensity and were classified as “satellite species”.

*Annulotrematoides parisellei* is classified as a “satellite species” in our sample, recorded parasitizing *C. macropomum* in Maranhão. This species was originally described parasitizing *S. brasiliensis* (Cohen et al., 2012) and its occurrence in *C. macropomum*, a new host, suggests an expansion of the host spectrum.

*Linguadactyloides brinkmanni* was originally described as parasitizing the gills of *C. macropomum* in the Amazon Basin (Thatcher & Kritsky, 1983). In Brazil, although early records focused on natural populations inhabiting Amazonian rivers (Fischer et al., 2003; Gonçalves et al., 2018), the occurrence of this monopisthocotylan in aquaculture systems has become increasingly frequent. Reports encompass both the original described host (*C. macropomum*) and the hybrid “Tambatinga” (*C. macropomum* ♀ × *P. brachypomus* ♂) (Table 1).

The first record of *L. brinkmanni* in hybrids in South America was documented in the state of Amapá, Brazil, where it exhibited low parasitic indices, with a prevalence of 4.9% and a mean intensity of 2.1 (Dias et al., 2012). In the present study, prevalence, intensity, and abundance values were found at intermediate levels when compared to other producing regions (e.g., Amapá and Rondônia) (Godoi et al., 2012; Dias et al., 2015a, b; Dias & Tavares-Dias, 2015). These findings corroborate previous literature describing *L. brinkmanni* as a persistent, though rarely dominant, component of parasitic communities in controlled environments.

*Colossoma macropomum* cultivated in the state of Acre presented the highest species richness of Monopisthocotyla, the highest number of infested hosts, and the highest parasite load, totaling 1,207 registered specimens, which confirms a scenario of high parasite pressure in this cultivation system. The high species richness, composed mainly of representatives of the genera *Anacanthorus*, *Mymarothecium*, *Linguadactyloides*, and *Notozothecium*, corroborates previous studies like Silva et al. (2022) and Rocha et al. (2018) which identified “Tambaqui” as one of the most susceptible species in the Serrasalmidae to monopisthocotylan infestation in natural and fish farming environments in the Amazon.

Species of Acanthocephala have been reported from fish reared in cultivated systems, which may cause serious injuries on the hosts (Valladão et al., 2020). The results of this study reveal the first record of *N. buttnerae* parasitizing *Piaractus brachypomus* in the state of Acre. The presence of species of *Neoechinorhynchus* is widely recognized as an indicator of ecological integrity and the maintenance of trophic interactions in aquatic ecosystems (Gouveia et al., 2021).

The highest prevalence of *N. buttnerae* in this study was recorded in *Colossoma macropomum* cultivated in the state of Maranhão, while significantly lower values were observed in *P. brachypomus* and in the hybrid *C. macropomum* ♀ x *P. brachypomus* ♂, from the state of Acre. This result corroborates previous studies that point to *C. macropomum* as the main host of *N. buttnerae* in fish farming systems in northern and northeastern Brazil, especially in intensive or semi-intensive farming, where there is greater availability of intermediate hosts, such as aquatic oligochaetes, which are fundamental to the parasite's biological cycle (Malta et al., 2001; Pavanelli et al., 2008; Santos et al., 2013; Jerônimo et al., 2017). According to Malta et al. (2001), environments with sediment rich in organic matter tend to support larger populations of oligochaetes, increasing the pressure of infection of *N. buttnerae* in “Tambaqui”.

The presence of trematodes in cultured fish serves as an important indicator of the ecological and sanitary conditions of aquatic environments. Owing to their complex heteroxenous life cycle, the occurrence of members of the class Trematoda, subclass Digenea, in the evaluated hosts necessarily implies the presence and proliferation of intermediate hosts, such as gastropod mollusks, within aquatic systems (Thatcher, 2006; Kohn et al., 2013).

*Dadaytrema oxycephalum* was first recorded in 1936 and is currently recognized for its wide distribution across South America, infecting fish of the orders Characiformes and Siluriformes in countries such as Brazil, Venezuela, Argentina, Peru, and Paraguay (Negreiros et al., 2024; Pantoja et al., 2012). Subsequent studies have expanded both the host spectrum and the geographic range of this species, highlighting its generalist capacity and preferential association with *Piaractus* (Campos et al., 2009; Oliveira & Tavares-Dias, 2016).

The present study reports, for the first time, the occurrence of the trematode *D. oxycephalum* parasitizing the hybrid *C. macropomum* ♀ x *P. brachypomus* ♂ in aquaculture systems in the state of Acre, Brazil. This finding expands the known host spectrum of the species and contributes significantly to the understanding of the helminth fauna associated with hybrid fish cultivated in the Amazon region. Parasitological descriptors observed for *D. oxycephalum* in *C. macropomum* ♀ × *P. brachypomus* ♂, particularly the low prevalence and reduced values of mean intensity and mean abundance (Table 2), indicate an isolated infection, which does not currently characterize the parasite as dominant within the parasitic community of this host species. Based on its low prevalence and abundance, this parasite can be considered a satellite species within the parasitic community of the evaluated hosts. This pattern is consistent with the literature, which describes *D. oxycephalum* as a trematode frequently associated with wild Amazonian fish, generally presenting low infestation levels in natural environments (Thatcher, 2006).

This finding reinforces that the movement of fish and hybrids between different regions can facilitate the spread of these parasites, promoting new geographical records. In addition, the literature highlights that the diversity of monopisthocotylans in farmed fish is often underestimated due to the lack of systematic studies in different regions and the intense exchange of biological material between fish farms (Silva et al., 2022; Tavares-Dias et al., 2022).

## Conclusion

The present study expands the known biodiversity and distribution of helminth parasites in South American aquaculture, providing new records for host-parasite associations and geographic locations in the states of Acre and Maranhão, Brazil.

This study highlights the intricate relationship between aquaculture practices and parasite ecology in tropical freshwater systems. The expansion of host–parasite records, combined with the identification of both dominant and emerging parasite species, reinforces the dynamic nature of these communities under cultivation conditions. These findings emphasize the urgent need for continuous parasitological monitoring and improved sanitary management to mitigate the risks of parasitic outbreaks, ensuring the economic and environmental sustainability of fish farming in the Neotropical Region.

Therefore, continuous parasitological monitoring, together with improved management practices such as controlling stocking densities, enhancing water quality, and reducing organic loads are essential to mitigate infection risks. Future studies should prioritize long-term assessments and comparative analyses between natural and artificial systems to better understand transmission dynamics and to support the development of sustainable and biosecure aquaculture in Brazil and across South America.

## Data Availability

All data are available within the text.
